# Genomics and inclusion of Indigenous peoples in high income countries

**DOI:** 10.1007/s00439-023-02587-5

**Published:** 2023-07-21

**Authors:** Kylie Gwynne, Shirley Jiang, Robertson Venema, Vita Christie, Tiffany Boughtwood, Marida Ritha, John Skinner, Nyesa Ali, Boe Rambaldini, Tom Calma

**Affiliations:** 1 Djurali Centre for Aboriginal and Torres Strait Islander Education and Research (Formerly Known as Poche Centre for Indigenous Health), Macquarie University, Walanga Muru Building, 6 First Walk, Sydney, NSW 2113 Australia; 2 University of Toronto, 27 King’s College Circuit, Toronto, Canada; 3 Australian Genomics, 50 Flemington Rd, Parkville, VIC 3052 Australia; 4 Murdoch Children’s Research Institute, Royal Children’s Hospital, 50 Flemington Rd, Parkville, VIC 3052 Australia

## Abstract

Genomics research related to Indigenous people has been at worst exploitative and at best, retrospectively on a journey to improve effective engagement of Indigenous individuals and communities. Genomics can positively impact all stages of clinical management, and to improve genomic effectiveness researchers aggregate genomic data from diverse global sub-populations, such as shared ancestry groupings, as people within these groupings will have a greater proportion of shared DNA traits. While genomics is already being used worldwide to improve lives, its utility and effectiveness has not been maximized for individuals with Indigenous ancestry. Several large datasets of human genetic variation have been made publicly available, of which the most widely used is the Genome Aggregation Database (gnomAD), but none of these databases currently contain any population-specific data for Indigenous populations. There are many reasons why Indigenous people have been largely left out of genomics research and, because of this, miss out on the benefits offered. It is also clear that if research is to be effective, it needs to be done ‘with’ and not ‘on’ Indigenous communities. This systematic review of the literature regarding Indigenous peoples (in high income countries) and genomics aims to review the existing literature and identify areas of strength and weakness in study design and conduct, focusing on the effectiveness of Indigenous community engagement.

**Supplementary Information:**

The online version contains supplementary material available at 10.1007/s00439-023-02587-5.

## Introduction

### Indigenous peoples and genomics

Genomics is the study of an individual’s genes (the genome), including interactions of those genes with each other and with the individual’s environment. It leverages the established understanding about genomic variation associated with wellness and disease to tailor medical treatment to the individual. It does this through targeted analyses of medically relevant information within an individual’s sequenced DNA. Genomics can positively impact all stages of clinical management: improving diagnosis, pinpointing appropriate preventive measures, and enabling therapeutic interventions (Rae et al 2017; National Research Council (US) 2011). To make genomics more effective, researchers aggregate genomic data from diverse global sub-populations, such as shared ancestry groupings, as people within these groupings will have a greater proportion of shared DNA traits (Robertson et al 2018). These groups can affect the nature and direction of the research undertaken and the interventions that are available to those groups.

While genomics is already being used worldwide to improve lives, its utility and effectiveness has not been maximized for individuals with Indigenous ancestry as these people are underrepresented in genomic reference databases (Petrovski and Goldstein 2016). Several large datasets of human genetic variation have been made publicly available, of which the most widely used is the Genome Aggregation Database (gnomAD), but these databases currently contain little, if any, population-specific data for Indigenous peoples (Karczewski et al 2020). The deficit of data affects equity of access to genomics for Indigenous peoples, who represent a culturally and linguistically diverse set of communities who are at greater risk of poorer health outcomes (Bilkey et al 2019). In the era of rapidly advancing medical care and technology, there is a serious risk that this inequity will widen the already prominent health and life expectancy gaps between Indigenous and non-Indigenous populations (Robertson et al 2018; Claw et al 2018).

Historically, the lack of study transparency in many scientific investigations has undermined trust and heightened concerns among Indigenous peoples about sharing personal health information (Middleton et al. 2019). While some people may have concerns regarding genetic research, such as genetic discrimination in employment, difficulty in obtaining insurance, or unconsented forensic use of genomic data in law enforcement (Claw et al 2018), Indigenous peoples may have additional concerns specific to their cultural, social and collective contexts. This may include allegations of genetic inferiority, threats to cultural beliefs, fear of exploitation for commercial purposes (e.g. drug development), inappropriate use in Native Title claims or exclusion from government assistance (Rae et al 2017; Robertson et al 2018). These concerns highlight the importance of adopting a collaborative approach when conducting genomic research involving Indigenous peoples; one which partners with Indigenous communities and incorporates Indigenous perspectives and culture into study design and conduct.

### Aim

This systematic review aims to gather the existing literature regarding Indigenous people and genomics and evaluate it based on the strength or weakness of the Indigenous community engagement of each study.

## Methods

### Literature search

An electronic literature search was conducted using the databases MEDLINE, CINAHL, and Aboriginal and Torres Strait Islanders Health Informit Database. The search strategy included two key concepts- Indigenous and genomics- and used the following search terms: “Indigenous”, “Aboriginal”, “Aborigines”, “Torres Strait*”, “First Nation”, “Metis”, “Inuit”, “American Indian”, “Native American”, “Alaska Native”; “genetics”, “genetic medicine”, “personalized medicine”, “genetic testing”, “genomics”, “genetic predisposition to disease”, “genetic diseases”, “biological specimen banks”. The search terms were adapted for other bibliographic databases in combination with database-specific filters, where applicable. Other inclusion criteria was English language, published since 2005, human studies only. The last search was performed on May 4th, 2020. The references of all included articles and related articles were hand searched to identify additional relevant studies.

**Fig. 1:**
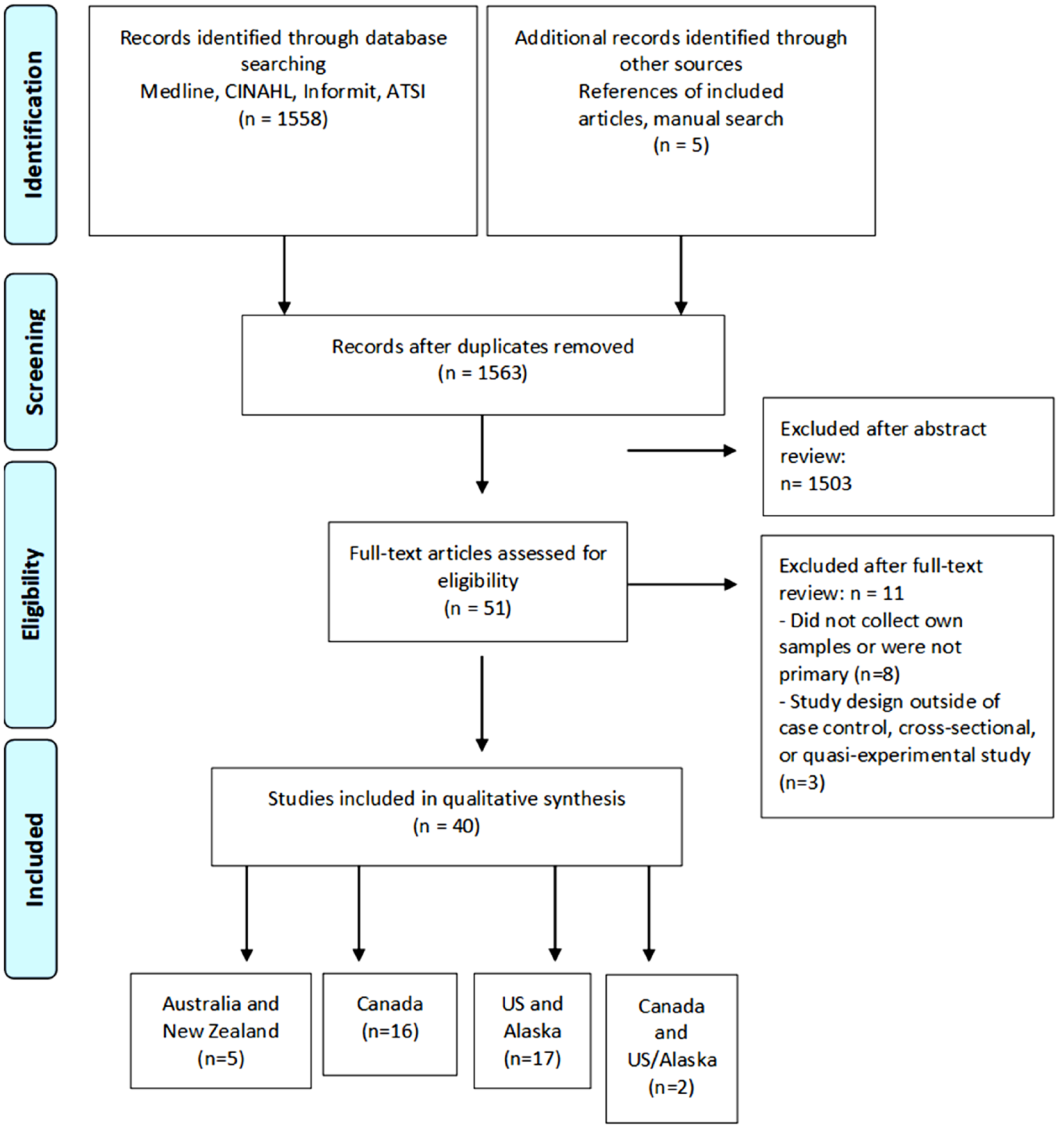
PRISMA flow diagram

A total of 1558 candidate articles were found in the initial database search (1415 from MEDLINE, 121 from CINAHL, 22 from the Informit database) and an additional five articles were identified through other sources. There were no duplicate articles. The abstracts of all articles were screened by two researchers (SJ, RV) and 51 articles were included for full-text review. Forty articles were included in the final analysis (see Fig. 1. PRISMA flow chart in the “Results” section).

### Study selection

The included studies were conducted in Indigenous populations in Australia, Canada, New Zealand, and the United States, studied a genetic basis for disease or treatment, and collected their own samples for genetic analysis. The geographical limitations reflect a recent qualitative review reporting similar experiences of Indigenous groups studied in these countries of genetic research (Aramoana et al. 2020). Studies were excluded if they: did not intend to study an Indigenous population or Indigenous status was determined in the analysis, used previously available samples (i.e. did not directly collect samples from participants), or if genetic analysis was performed for the purpose of forensic biology or evaluation of genetic sequencing techniques. Only case control, cross-section, and quasi-experimental studies were included. The inclusion of each article was reviewed by two researchers (SJ, RV) and consensus was reached on consultation with a senior researcher (KG).

### Risk of bias assessment

Included studies were scored using the Joanna-Briggs Institute Critical Appraisal Checklist for case control, cross-section, or quasi-experimental studies.

### Data abstraction and analysis

Pre-determined piloted forms were used to extract data independently by two researchers (SJ, RV) and included: population group, location, sample size, study design, consent and recruitment process, research governance, data dissemination, genetic analysis procedures, and a community engagement score (CES, discussed in further detail below). Given the scope of the study, it was not possible to contact the original investigators of any study.

The Community Engagement Score (CES) is a measure of community involvement and ethical approaches towards research with Indigenous populations. It is derived from the principles of the National Health and Medicine Research Council (NHMRC) of Australia on ethical conduct in Aboriginal and Torres Strait Islander health research. (Jamieson et al. 2012; National Health and Medical Research Council of Australia 2018). The guidelines have been modified to make up five separate categories: community-centered, partnership and governance, capacity-building, flexibility and cultural considerations, and respect for communities. Each study was assessed against each category and scored 1 for yes and 0 for no. The study was then assigned a score out of five based on meeting these components, with a description of ethical measures taken. Other information was also extracted, including population, year of publication, study design, appraisal score, gene technique, and study findings. Where multiple publications were derived from the same study, each publication was scored based on its own reporting. This information is summarized in Supplementary Table 1: Overview of Included Studies by Community Engagement Methods.

Best practice examples of community engagement methods were summarized among all the included studies. These examples are either extracted from original articles or associated methodological papers or commentaries of the specific study in question, where applicable.

### Study characteristics

40 articles were included in the final analysis. The study populations were primarily North American, based in US and Alaska (*n* = 17), Canada (*n* = 16), or joint between Canada and the US (*n* = 2). There were also 5 articles from Australia (*n* = 3) and New Zealand (*n* = 2). Of the included publications, there was overlap in study populations found in 16 articles, in the form of updated report on longitudinal cohorts. As such, there are only 31 unique research studies included. However, each article was analyzed based on its own report and associated methods paper, if applicable.

Of the 40 included articles, there were 21 cross-sectional studies, 18 case control studies, and 1 quasi-experimental study. The average score by the Joanna-Briggs Institute Critical Appraisal Checklist was 7.14 for cross sectional studies, 8.88 for case control studies and 8 for quasi-experimental studies. Genetic sequencing methods used were targeted genotyping by Polymerase Chain Reaction (PCR) (*n* = 31, 77%) and genome-wide association studies (*n* = 9, 22%). The average CES score was 2.5, with reported actions towards community centeredness in 8 articles (20%), partnership and governance in 14 articles (35%), capacity-building in 6 articles (15%), flexibility and cultural considerations in 9 articles (22%), and respect in 12 articles (35%).

## Results

### Findings

Data from the methods of included articles were used to provide key examples of best practice in the following Table 1 Community Engagement Areas.

**Table 1:** Community engagement areas

Community engagement area	Description	*N* (%)	Examples of best practice
Community-centered	Addressing a priority health issue determined by the community, by working closely with the community	8 (20%)	• Sought consultation with the community to determine priority health issue (Fohner et al. 2013; Fohner et al. 2015; Scally et al. 2017) • Community-initiated projects whereby community members approached researchers with concern of disease (Arbour and Cook 2006; Asuri et al. 2018)
Partnership and governance	Conducting research within a mutually respectful partnership, with Indigenous community members in key leadership positions	14 (35%)	• Establishing an Indigenous advisory group comprising community members (Arbour and Cook 2006; Fohner et al. 2013; Gray et al. 2017; Binnington et al. 2012; McWhirter et al. 2014, 2014; Murdoch et al. 2012; Scally et al. 2017; Voruganti et al. 2010; Zhu et al. 2013) • Establishing a long-term relationship through creation of a long-term cohort using community-based participatory research (CBPR) methods (Asuri et al. 2018; Fohner et al. 2013; Larcombe et al. 2015; Larcombe et al. 2017; McWhirter et al. 2014) • Extensive community consultation and consent obtained from both local health board, community leaders, and community members to ensure comprehensive buy-in for the project (Arbour and Cook 2006; Asuri et al. 2018; Gray et al. 2017; McWhirter et al. 2014)
Capacity-building	Capacity building as a key focus of the research partnership	6 (15%)	• Employing local community members to assist in research for skill development and employment opportunities (Arbour and Cook 2006; Asuri et al. 2018; McWhirter et al. 2014; Voruganti et al. 2010) • Research collaboration facilitated improved diagnosis, treatment, or management of the disease studied including increased public health interventions, screening and treatment of carriers, accessibility of treatment measures (Arbour and Cook 2006; McWhirter et al. 2014) • Research collaboration created or supported existing community health programs including education with healthcare professionals, food-based programs, promoting health lifestyles and access to healthcare (Arbour and Cook 2006; McWhirter et al. 2014)
Flexibility and cultural considerations	Flexibility in study implementation to respect Indigenous culture	9 (22%)	• Adapted information materials by working with community representatives to create culturally acceptable and informative materials (Asuri et al. 2018, p. 201; McWhirter et al. 2014) • Consent process was performed with culturally appropriate materials and local interpreters to ensure understanding and that all questions were answered (Gray et al. 2017; McWhirter et al. 2014). Interpreter present for consent process (El-Gabalawy et al. 2011; Scally et al. 2017; Voruganti et al. 2010) • Adaptation of protocol to community ways of knowing and being e.g. story-telling (McWhirter et al. 2014), hunting season (Voruganti et al. 2010), and using whakapapa to construct heritage (Cameron-Christie et al. 2018)
Respect	Respecting communities’ past and present experience of research	12(35%)	• Agreement with community in the form of a research agreement or Memorandum of Understanding (Anderson et al. 2015; Murdoch et al. 2012; Scally et al. 2017) • Agreement with participants as part of consent process, sample use, and subsequent studies (Arbour and Cook 2006; Asuri et al. 2018; Cameron-Christie et al. 2018; McWhirter et al. 2014) • Result disseminated regularly to community members (Arbour and Cook 2006; Asuri et al. 2018; Scally et al. 2017), and/or prior to publication to seek approval from elders or community members (Anderson et al. 2015; Asuri et al. 2018; McWhirter et al. 2014; Voruganti et al. 2010) • In decisions related to previous negative Indigenous experiences with genetic research, advice was sought from the community on how to proceed (McWhirter et al. 2014, 2014) • Ensure that research protocols respect community wishes, e.g. not to collect origin or relatedness data (Fohner et al. 2013; Larcombe et al. 2015)

### Findings and discussion

Our review identified five areas for improvement when addressing the gap in Indigenous genomic research engagement.

#### Area 1: Research should address a priority health issue determined by the community

Past research has often been labelled as extractive: the main objective of a study was to answer a scientific question of interest to the researcher, and participants were viewed as experimental subjects utilized to answer the question (Arbour and Cook 2006). Even when a health issue leads to high burden of disease in the community, it is prudent to ensure that it is of importance to the community (Jamieson et al. 2012).

Some studies were designed using codesign methodology, including community consultation to determine whether the research topic was a priority health issue (Fohner et al. 2013; Fohner et al. 2015; Scally et al. 2017) and to clarify which specific areas to address with research, such as knowledge translation to the community (McWhirter et al. 2014). In one study, researchers were approached by community members with specific concern for a prevalent disease, leading to a community-driven study and fruitful research partnership (Arbour and Cook 2006; Asuri et al. 2018).

#### Area 2: Indigenous governance is needed to conduct research within a mutually respectful partnership

A mutually respectful partnership between researchers and the community should be formed. While community members can be study participants, they are also uniquely poised to advise on research issues, process, and resulting priority interventions (McWhirter et al. 2014). Approval sought for research should not be limited to certain groups or a point in time. Researchers should seek agreement to conduct research beyond that of local health authorities, institutional review boards, or community leaders; rather researchers should seek wide-ranging approval of community members who the results may also impact (McWhirter et al. 2014). Approval to initiate a research project should also include a mechanism to make any post-approval changes (Jamieson et al. 2012).

Many researchers in reviewed articles established an Indigenous Advisory Group made of community members to advise on the research process (Arbour et al. 2008; Fohner et al. 2013; Gray et al. 2017; Binnington et al. 2012; McWhirter et al. 2014; Murdoch et al. 2012; Scally et al. 2017; Voruganti et al. 2010; Zhu et al. 2013). Where a prospective cohort study was initiated, a long-term relationship was emphasized between researchers and community members (Asuri et al. 2018; Fohner et al. 2013; Larcombe et al. 2015; Larcombe et al. 2017; McWhirter et al. 2014). Extensive community consultation and consent was obtained from local health boards, community leaders, and community members to ensure comprehensive community approval for the research project (Arbour et al. 2008; Asuri et al. 2018; Gray et al. 2017; McWhirter et al. 2014).

#### Area 3: Capacity-building is a key focus of the research partnership

This area seeks to provide benefits to the community beyond those of scientific progress. Benefits provided can include skill development, in-kind benefits, or public health interventions. Where possible, research should provide opportunities for additional benefits to participants and/or the broader community.

There were several instances where local community members were employed to assist in research for skill development and employment. One study (McWhirter et al. 2014) employed local community members to facilitate consent processes which improved cultural appropriateness and decreased the power dynamic during participant enrolment. Several researchers (Arbour and Cook 2006; Voruganti et al. 2010) employed Indigenous students or local community members as research assistants, who helped with translation. Research collaborations also provided community interventions such as interactive sessions with health professionals, community activities, and increased access to healthcare (Arbour and Cook 2006; McWhirter et al. 2014).

#### Area 4: Flexibility in study implementation to respect Indigenous ways of doing and knowing

Studies based on community feedback and recognition of Indigenous ways of doing and knowing can improve the scientific rigor of the research (Jamieson et al. 2012). This principle arose from previous studies that were based on non-Indigenous scientific standards without consideration of Indigenous culture (Dodson and Williamson 1999).

In our review, several researchers partnered with community members to create information materials in a culturally acceptable and informative manner (Arbour and Cook 2006; Asuri et al. 2018; McWhirter et al. 2014), and the consent process often included an interpreter (El-Gabalawy et al. 2011; Gray et al. 2017; Scally et al. 2017; Voruganti et al. 2010). In data dissemination, one study provided results to the community in a video format with cultural reference points and traditional stories as a framework for the health message (McWhirter et al. 2014).

Research rigor has also been improved when study methodology operates within Indigenous culture. For example, one study (Voruganti et al. 2010) planned around hunting season, ensuring the timing of the research did not clash with periods when the community were transient in nature, and one study (Cameron-Christie et al. 2018) used traditional genealogy practices (’whakapapa’) to construct heritage.

#### Area 5: Respecting communities’ past and present experience of research

Past genetic research has led to many transgressions against Indigenous peoples worldwide including propagating racial stereotypes, unauthorized genetic analysis, unreturned genetic samples, and using results to deny Indigenous culture (Taniguchi et al. 2012). Thus, an understanding of previous exploitative research in the context of colonization is required to build new research partnerships based on respect. Researchers should establish clear expectations in written agreements where possible, ensure community ownership of all intellectual and biological assets, and apply iterative changes to the study protocol based on community feedback (Jamieson et al. 2012).

We found that many researchers formed a Memorandum of Understanding with the community as a way of ensuring agreement in research (Anderson et al. 2015; Murdoch et al. 2012; Scally et al. 2017). Further, participants’ agreement was ensured as part of initial consent, sample use, and subsequent studies if samples were to be re-analyzed for other purposes (Arbour et al. 2008; Asuri et al. 2018; Gray et al. 2017; McWhirter et al. 2014). Results were disseminated regularly to communities (Asuri et al. 2018; Scally et al. 2017) and approval was sought from community members prior to publication (Anderson et al. 2015; Asuri et al. 2018; McWhirter et al. 2014; Voruganti et al. 2010). Where contentious past research was needed for comparison, advice was sought from Indigenous leadership on how to proceed, demonstrating respect for past transgressions (McWhirter et al. 2014). Finally, some research protocols demonstrated respect for community wishes and cultural beliefs, for example one study (Fohner et al. 2013) avoided collecting relatedness data and another (Larcombe et al. 2015) avoided analysis of geographical or evolutionary origins of participants.

### Limitations

Currently limited research exists that is led by Indigenous people about Indigenous genomics, indicating a limitation in genomics research. Therefore, it is essential that extensive further research is performed including Indigenous people in this field. From the literature extracted, it is evident that some of the research performed on Indigenous genomics was done so in an unethical manner and would not be validated by majority of the human research ethics committees globally.

## Conclusion

This systematic review has identified several areas of opportunities and improvements necessary to address the large gap in successfully conducting research in Indigenous genomics. The review has shown that many of the areas of opportunities lie around the need to conduct research in a manner that should address a priority health issue that is deemed so by Indigenous communities; that there is the need for Indigenous governance to ensure research is conducted via a mutual partnership that is respectful; and that flexibility is essential in study implementation to demonstrate respect for culturally appropriate Indigenous ways of doing and knowing and ensuring respect for communities’ experiences both past and present.

## Supplementary Information

Below is the link to the electronic supplementary material.

Supplementary file1 (DOCX 74 KB)

## Data Availability

The datasets generated during and/or analyzed during the current study are available from the corresponding author on reasonable request.
