# Supplementary material for: Genomics and inclusion of Indigenous peoples in high income countries
Source: Hum Genet. 2023 Jul 21;142(9):1407–16. doi: 10.1007/s00439-023-02587-5 (PMC10449672; doi:10.1007/s00439-023-02587-5)
Supplement: Supplementary file 1 — Supplementary file1 (DOCX 74 KB) [file 439_2023_2587_MOESM1_ESM.docx]

**Supplementary Table 1. Overview of Included Studies by Community Engagement Methods.**

| **Country** | **Cohort** | **Study** |  |  | **CES**  **score (1-5)** | **JBI score (0-10)** | **Methods for Community Engagement** | **Results** | |
| --- | --- | --- | --- | --- | --- | --- | --- | --- | --- |
|  |  | **Authors** | **Year** | **Study design, n** |  |  |  | **Genetic study** | **Findings/Impact** |
| Australia | Aboriginal women of Arnhem Land (North Territory) | McWhirter et al. (McWhirter et al., 2014) | 2014 | Case control, n=92 (30 cases) | 5 | 10^CC^ | Formed Indigenous Reference Group (IRG) to advise on all study aspects.  Option to consent for ongoing/future use of samples.  Results disseminated in storytelling format in keeping with local culture.  Longitudinal relationship between researchers and community.  Methods paper available.(McWhirter et al., 2012) | GWAS (microarray) | Vulvar cancer and dysplasia was not significantly associated with homozygosity of SNPs in this population. |
|  | Aboriginal communities in Northern Australia | Gray et al. (Gray et al., 2017) | 2017 | Case control, n=1263 (398 cases) | 4 | 10^CC^ | “Opt-in” design.  Formed project steering committee and subcommittees with Indigenous membership, including an Aboriginal governance committee.  Developed culturally appropriate consent materials. | GWAS (microarray) | Rheumatic Heart Disease was associated with a major genetic risk factor (HLA_DQA1-DQB1) by pathogenic molecular mimicry in this population. |
|  | Aboriginal Australians of Martu ancestry from Western Desert (Western Australia) | Anderson et al. (Anderson et al., 2015) | 2015 | Cross-sectional, n=405 | 2 | 8^CS^ | Consulted local Aboriginal Health Service, including elders from communities. | GWAS  (microarray) | No genome-wide associations achieved significance for diabetes and obesity in this population. |
| New Zealand | Māori | Cameron-Christie et al. (Cameron-Christie et al., 2018) | 2018 | Cross-sectional, n=12 | 2 | 6^CS^ | Constructed pedigree using whakapapa (recounting of lineage and familial relationships) by family members. | GWAS (whole exome sequencing and microarray) | No reproductive isolation appears to account for genetic risk to explain the high incidence of biliary atresia in this population. |
|  | Genetics of Gout, Diabetes, and Kidney Disease in Aotearoa New Zealand study  NgatiPorou iwi of Tairawhiti  Pukapuka Island | Krishnan et al. (Krishnan et al., 2018) | 2018 | Cross-sectional, n=2286 | 1 | 6^CS^ | Recruited in collaboration with local health provider and charitable group. | GWAS  (microarray) | Favorable adiposity, defined as higher BMI with lower incidence of type 2 diabetes mellitus, is associated with the A-allele (rs373862828) in this population. |
| Canada | First Nations families in British Columbia | Asuri et al. (Asuri et al., 2018) | 2017 | Case control, n=105 (44 cases) | 5 | 10^CC^ | Community-based, “DNA on loan” design.  Project initiated at traditional potlach, overarching research consultation process through community leaders, and continued development through community meetings.  Capacity building by using undergraduate First Nations students as research assistants.  Information dissemination by local newsletter more likely to reach individuals and pamphlets designed with research participant feedback.  Longitudinal relationship with research committees, local health units and participants and their families.  Methods paper available. (Arbour & Cook, 2006) | GWAS (microarray) | Primary Biliary Cholangitis (PBC) may be associated with multiple SNPs which converge on a signaling pathway, in association with other environmental and epigenetic factors, to increase risk of PBC in this population. |
|  | Gitxsan First Nation  (British Columbia) | Arbour et al. (Arbour et al., 2008; Arbour & Cook, 2006) | 2008 | Cross-sectional, n=124 | 5 | 8^CS^ | Community-initiated based on high incidence and to advise management, “DNA on loan” design.  Local research advisory committee with community members, medical personnel, and local health authority.  Capacity building by individualizing care for identified mutation carriers, installing community medical devices, and planned education strategies. | Targeted sequencing | Long QT syndrome prevalence, as indicated by prolonged corrected QT interval, may be associated with KCNQ1 V205M missense mutation in this population. |
|  |  | Swanye et al. (Swayne et al., 2017) | 2007 | Cross-sectional, n=18 | 2 | 8^CS^ | Subset of Arbour et al.(Arbour & Cook, 2006) | Targeted sequencing | Ankyrin B syndrome and structural heart disease associated with novel genetic variant (ANK2 membrane-binding domain) in this population. We note that the methods paper associated with this study scored a 5. This paper only scored a 2 because they did not report on the community engagement in the research. |
|  | Dene First Nation  (Manitoba) | Larcombe et al.(L. A. Larcombe et al., 2008) | 2008 | Cross-sectional, n=194 | 2 | 8^CS^ | Addresses disease relevant to community.  Ethical approval sought from participating First Nations communities. | Targeted genotyping (PCR) | Tuberculosis risk may be associated with SNPs in genes encoding key immune modulators (MCP-1, VDR, IFN-y, IL-6 alleles). |
|  |  | Larcombe et al.(L. Larcombe et al., 2015) | 2015 | Quasi-experimental, n=54 | 4 | 8^QE^ | Followed ethical principles of “ownership, access, control and possession (OCAP^[[1]](#footnote-1)^)” in design.  Formed community partnership to identify relevant issues.  Protocol and approach determined with community consultation.  Respected community wishes of avoiding conjecture regarding geographical or evolutionary origin. | Targeted genotyping (PCR, RFLP) | Vitamin D supplementation to boost innate immune response may be associated with SNPs in vitamin D metabolism and associated levels of host defense peptide LL-37; in this population, it may not be beneficial to provide vitamin D supplementation for improved immune response. |
|  | Cree First Nation  Dene First Nation  (Manitoba) | Larcombe et al. (L. A. Larcombe et al., 2017) | 2017 | Cross-sectional, n=2700 | 4 | 8^CS^ | Reports similar protocol as Larcombe et al. 2015 (L. Larcombe et al., 2015), with addition of Cree First Nation participants. | Targeted genotyping (PCR) | Tuberculosis prevalence may be associated with certain HLA types regulating inflammatory response in this population. |
|  | Cree First Nation  Dene First Nation  Ojibway First Nation  (Manitoba) | Braun et al. (Braun et al., 2013) | 2013 | Case control, n=168 (93 cases) | 2 | 8^CC^ | Addresses disease relevant to community.  Ethical approval sought from participating First Nations communities. | Targeted genotyping (PCR) | Tuberculosis prevalence may be associated with certain centromeric haplotypes (KIR) regulating immune response in this population. |
|  | Cree First Nation  Ojibway First Nation  (Manitoba) | Oen et al. (Oen et al., 2005) | 2005 | Case control, n=127 (82 cases) | 1 | 9^CC^ | Addresses disease relevant to community. | Targeted genotyping (PCR) | Rheumatoid arthritis risk may be decreased by certain immunomodulatory genotypes (IL-10). |
|  |  | El-Gabalawy et al.(El-GABALAWY et al., 2009) | 2009 | Case-control, n=473 (266 cases) | 2 | 9^CC^ | Addresses disease relevant to community.  Approval by Tribal Band Councils. | Targeted genotyping (PCR) | Rheumatoid arthritis risk may be associated with specific genotypes in this population (DRB1*0901, SE/DRB1*0901). |
|  |  | El-Gabalawy et al.(El-Gabalawy et al., 2011) | 2011 | Case control, n=823 (333 cases) | 3 | 8^CC^ | Addresses disease relevant to community.  Approval by Tribal Band Councils.  Consent process in language of participants’ choice. | Targeted genotyping (PCR, mass spectrometry chip) | Rheumatoid arthritis risk may be modulated by non-HLA genes (MMEL1-TNFRSF14 and TRAF1-C5) in this population. |
|  |  | Hitchon et al. (Hitchon et al., 2012) | 2012 | Case control, n=1152 (448 cases) | 2 | 10^CC^ | Addresses disease relevant to community.  Approval by Tribal Band Councils. | Targeted genotyping (PCR, mass spectrometry chip) | Rheumatoid arthritis prevalence may be associated with vitamin D polymorphisms (Fok1 SNP) in this population. |
|  | Cree First Nation  Ojibway First Nation  Oji-Cree First Nation  Metis peoples  (Manitoba) | Rempel et al.(Rempel et al., 2011) | 2011 | Case control, n=179 (86 cases) | 3 | 8^CC^ | Addresses disease relevant to community.  Preformed in discussion with community leaders and health research committee. | Targeted genotyping (PCR) | Killer immunoglobulin-like receptor (KIR) cluster profiles may represent an immune selection process which confers susceptibility to disease in the current population. |
|  | Ste Theresa Point First Nation  Norway House Cree Nation  (Manitoba) | Murdoch et al. (Murdoch et al., 2012) | 2012 | Case control, n=625 (340 cases) | 3 | 9^CC^ | Followed Canada Institutes of Health Research guidelines for conducting research involving Canada’s Indigenous population. (<https://cihr-irsc.gc.ca/e/29134.html>)  Established advisory committee to provide oversight.  Formal research agreements with communities. | Targeted genotyping (mass spectrometry chip) | Low prevalence of inflammatory bowel disease in this population (despite high prevalence of other autoimmune disease) may be associated with variants in immune response and bacteria handling mechanisms that confer protection against IBD. |
|  | Oji-Cree of Sandy Lake  (Ontario) | Pollex et al. (Pollex et al., 2006) | 2006 | Cross-sectional, n=728 | 5 | 8^CS^ | Community-initiated for prevalent disease.  Community-led governance of project.  Capacity-building through multiple health programs surrounding disease including radio show, school-based activity, home visit program; hiring community members to lead programs; community feedback.  Ongoing longitudinal relationship between researchers and community.  Methods paper available.(Kakekagumick et al., 2013) | Targeted genotyping (PCR) | Type 2 diabetes mellitus risk may be increased synergistically by hypertriglyceridemic waist and lipid metabolism gene (HNF1a) in this population, with higher prevalence found in men. |
|  | Nunavik (Quebec) | Zhou et al. | 2015 | Cross-sectional, n=113 | 2 | 4^CS^ | Addresses disease relevant to community. | GWAS (microarray, targeted exome sequencing) | Fatty acid metabolism related genes have higher number and frequency of deleterious variants in this population. |
|  | Inuit women  (Northe  rn Quebec) | Metcalfe et al.(Metcalfe et al., 2013) | 2013 | Cross-sectional, n=548 | 2 | 6^CS^ | Addresses disease relevant to community.  Ethical approval sought from local health authority. | Targeted sequencing | HPV infection risk may be associated with HLA-G allele in this population. |
| United States | Alaska Natives from the Bristol Bay region (Alaska) | Binnington et al.(J. Binnington et al., 2012) | 2012 | Cross-sectional, n=400 | 3 | 8^CS^ | Addresses disease relevant to community.  Established community advisory board and sought approval from tribal council.  Worked with locals to develop language relatable to the study population.  See methods paper (Renner et al., 2013). | Targeted genotyping | Elevated tobacco-related disease rates may be related to high CYP2A6 activity in the Alaska Native population due to potentially greater tobacco carcinogen activation. |
|  |  | Zhu et al.(Zhu et al., 2013) | 2013 | Cross-sectional, n=400 | 3 | 6^CS^ |  | Targeted genotyping | Nicotine intake and body weight in tobacco users may be modulated by genetic variation in CHRNA5-A3-B4 in this population. |
|  | Yup’ik people recruited as part of CANHR study (Alaska) | Aslibekyan et al.(Aslibekyan et al., 2013) | 2013 | Cross-sectional, n=1136 | 5 | 7^CS^ | Established a joint research committee with local leaders. Followed CBPR principles.  Set research priorities with community input.  Built long-term relationships with communities.  Methods were informed by community consultation.  Developed health promotion efforts as part of research process.  Share results with community and collaboratively develop future research questions. | GWAS | Metabolic disease in Alaska Natives may be predicted by newly-identified loci including lipid loci on chromosomes 19 and 11. |
|  |  | Klimentidis et al.(Klimentidis et al., 2014) | 2014 | Cross-sectional, n=1144 | 5 | 7^CS^ |  | Targeted genotyping | Type 2 diabetes risk may be modified by CDKAL1 and HHEX, which may contribute to an understanding of this population’s relatively low rate of diabetes. |
|  | Alaska Natives recruited from Alaska Native Medical Center (Alaska) | Ferucci et al.(Ferucci et al., 2011) | 2011 | Cross-sectional, n=71 (57 of whom were Alaska Natives) | 1 | 6^CS^ | Ethical approval sought from local health authority. | Targeted genotyping (PCR) | In patients with autoimmune hepatitis, azathioprine toxicity or disease remission were not associated with TPMT genotype. |
|  | Alaska Natives recruited from Alaska Native Medical Center (Alaska) AND Yup’ik people recruited as part of CANHR study (Alaska) | Fohner et al.(A. E. Fohner et al., 2015) | 2015 | Cross-sectional, n=730 | 2 | 7^CS^ | Research question was developed with community input.  Sought approval from each tribal council, as well as the Indian Health Service and local institutional review board. | Targeted sequencing and genotyping | Genetic variants associated with lower warfarin requirements, including VKORC1 and CYP450, were more prevalent in the Alaska Native population than in the general population. |
|  | Alaskan Eskimos of Norton Sound recruited as part of the GOCADAN study (Alaska) | Voruganti et al.(Voruganti et al., 2010) | 2010 | Cross-sectional, n=761 | 5 | 8^CS^ | Research question of importance to community and was developed with community input.  Committee formed to review study progress and publications.  Permission was sought from individual village councils for recruitment.  Cultural factors, such as timing of hunting/fishing were considered.  Field work was contracted to local agency, and locals were employed as translators where study investigators were personally involved. See methods paper (Howard et al., 2005). | Targeted genotyping and short tandem repeat analysis | Plasma fatty acid distribution was found to be strongly influenced by genetic factors including APOJ, LPL and TNFRSF10B. |
|  | American Indians (North Dakota) | Seal et al.(Seal et al., 2011) | 2011 | Cross-sectional, n=49 | 2 | 8^CS^ | Addresses disease relevant to community.  Approval sought from local health authority and tribal council. | Targeted genotyping (PCR) | Infant birth weight and formula intake are not associated with the A allele of the FTO gene. |
|  | Turtle Mountain Band of Chippewa (North Dakota) | Best et al.(Best et al., 2013) | 2013 | Case control, n=410 (140 cases) | 1 | 10^CC^ | Addresses disease relevant to community. | Targeted genotyping (microarray and PCR) | Pre-eclampsia risk was associated with two variants of the CRP gene. |
|  |  | Best et al.(Best et al., 2012) | 2012 | Case control, n=196 (66 cases) | 1 | 10^CC^ | Approval sought from local health authority and tribal council. | Targeted genotyping (PCR) | Risk of severe pre-eclampsia was associated with a variant of the CRP gene. |
|  | Northern Plains American Indians (South Dakota) | Best et al.(Best et al., 2017) | 2017 | Case-control, n=323 (108 cases) | 1 | 10^CC^ | Addresses disease relevant to community. | Targeted genotyping (PCR) | Asthma risk was associated with variants at 5q22.1 and 17q21. |
|  | Choctaw Nation (Oklahoma) | Weiner et al.(M. Weiner et al., 2009) | 2009 | Case-control, n=21 (11 cases) | 1 | 8^CC^ | Approval sought from tribal IRB. | Targeted sequencing | Alzheimer’s Disease risk was associated with having apolipoprotein Eε4 alleles in this population. There was no association between degree of Indian heritage and the presence of this allele. |
|  | Choctaw Nation (Oklahoma) | Weiner et al.(M. F. Weiner et al., 2011) | 2011 | Case-control, n=117 (39 cases) | 1 | 9^CC^ | Addresses disease relevant to community.  Approval sought from tribal IRB. | Targeted genotyping (PCR) | Fewer American Indian patients with Alzheimer’s disease had Apolipoprotein Eε4 alleles present than did their Caucasian counterparts. This suggests that heredity may be a smaller contributor to Alzheimer’s risk in the American Indian population. |
|  | Confederated Salish and Kootenai Tribes (Montana) | Fohner et al.(A. Fohner et al., 2013) | 2013 | Cross-sectional, n=187 | 3 | 7^CS^ | Research question of importance to community and was developed with community input.  Approval sought by tribal council and tribal health authority.  Study methods were shaped by cultural considerations. | Targeted sequencing | Drug metabolism, and as a result, dosing, may be different in the American Indian population because of a difference in CYP450 allele frequency than the general population. |
|  | Native Americans who have undergone renal transplantation (Arizona) | Chakkera et al.(Chakkera et al., 2013) | 2013 | Case control, n=48 (24 cases) | 1 | 9^CC^ | Addresses disease relevant to community. | Targeted genotyping (PCR) | Therapeutic trough levels of the immunosuppressive drug tacrolimus were achieved with lower doses in the Native American population compared to Caucasian controls. This corresponded to an increased prevalence of genotypes associated with lower tacrolimus dosing. |
|  | Mission Indians (California) | Ehlers et al.(Ehlers et al., 2010) | 2010 | Cross-sectional, n=620 (genetic study n=381) | 2 | 8^CS^ | Addresses disease relevant to community.  Study methods were shaped by cultural considerations. | GWAS | Substance dependence-related traits were associated with regions of chromosomes 6, 9 and 10 were associated with substance dependence-related traits in this population. |
|  |  | Ehlers et al.(Ehlers et al., 2012) | 2012 | Cross-sectional, n=1182 |  | 6^CS^ |  | Targeted genotyping | Alcoholism risk was associated with variants of the alcohol dehydrogenase 1B gene. Different variants were protective against alcoholism in the Native American vs Mexican American populations studied. |
| US/Canada | Indigenous Canadians from Manitoba AND Alaska Natives | Ferucci et al.(Ferucci et al., 2013) | 2013 | Case control, n=293 | 1 | 6^CC^ | Addresses disease relevant to community. | Targeted genotyping (PCR) | In people with Rheumatoid Arthritis and their first-degree relatives, there was no association between shared epitope allele and the presence of anti-PAD4 antibodies. This mirrors findings in other ethnic groups. |
|  | Indigenous Canadians from Manitoba AND Alaska Natives | Scally et al.(Scally et al., 2017) | 2017 | Case control, n=696 | 3 | 7^CC^ | Research question of importance to community and was developed with community input.  Followed CBPR principles.  Approval sought by tribal council and tribal health authority.  Regular knowledge-translation activities were undertaken.  See supplemental methods. | Targeted genotyping (PCR) | The increased risk of ACPA^+^ Rheumatoid Arthritis in the Indigenous North American population may be related to an HLA-DRB1*14:02 variant, which broadens the capacity for presentation of citrullinated and native self-peptide presentation. |

1. Sanctioned by the First Nations Information Governance Committee, Assembly of first Nations. Ottawa: National Aboriginal Health Organization; 2007 [↑](#footnote-ref-1)
